# Identification of the Genome Segments of Bluetongue Virus Type 26/Type 1 Reassortants Influencing Horizontal Transmission in a Mouse Model

**DOI:** 10.3390/v13112208

**Published:** 2021-11-02

**Authors:** Houssam Attoui, Baptiste Monsion, Bernard Klonjkowski, Stéphan Zientara, Peter P. C. Mertens, Fauziah Mohd Jaafar

**Affiliations:** 1 UMR1161 Virologie, INRAE, Ecole Nationale Vétérinaire d’Alfort, Anses, Université Paris-Est, F-94700 Maisons-Alfort, France; baptiste.monsion@vet-alfort.fr (B.M.); bernard.klonjkowski@vet-alfort.fr (B.K.); stephan.zientara@vet-alfort.fr (S.Z.); 2 School of Veterinary Medicine and Science, Sutton Bonington Campus, University of Nottingham, Leicestershire LE12 5RD, UK; peter.mertens@nottingham.ac.uk

**Keywords:** bluetongue, bluetongue virus, BTV, BTV-1, BTV-26, horizontal transmission, vector independent transmission, reassortment, IFNAR^(−/−)^ mice

## Abstract

Bluetongue virus serotypes 1 to 24 are transmitted primarily by infected *Culicoides* midges, in which they also replicate. However, “atypical” BTV serotypes (BTV-25, -26, -27 and -28) have recently been identified that do not infect and replicate in adult *Culicoides*, or a *Culicoides* derived cell line (KC cells). These atypical viruses are transmitted horizontally by direct contact between infected and susceptible hosts (primarily small ruminants) causing only mild clinical signs, although the exact transmission mechanisms involved have yet to be determined. We used reverse genetics to generate a strain of BTV-1 (BTV-1 RG_C7_) which is less virulent, infecting IFNAR^(−/−)^ mice without killing them. Reassortant viruses were also engineered, using the BTV-1 RG_C7_ genetic backbone, containing individual genome segments derived from BTV-26. These reassortant viruses were used to explore the genetic control of horizontal transmission (HT) in the IFNAR^(−/−)^ mouse model. Previous studies showed that genome segments 1, 2 and 3 restrict infection of *Culicoides* cells, along with a minor role for segment 7. The current study demonstrates that genome segments 2, 5 and 10 of BTV-26 (coding for proteins VP2, NS1 and NS3/NS3a/NS5, respectively) are individually sufficient to promote HT.

## 1. Introduction

Bluetongue is an arboviral disease of ruminants, caused by *Bluetongue virus* (BTV), the type species of genus *Orbivirus*. Orbiviruses are transmitted between susceptible hosts by the bites of vector competent, hematophagous arthropods, although vectors have not been identified for all orbiviruses [1]. Most strains of BTV are transmitted between their ruminant hosts via the bites of infected adult female midges (*Culicoides* spp.). They can infect a number of competent *Culicoides* species [2], including colony derived adult *Culicoides sonorensis* (via an oral or intrathoracic route), or a *C. sonorensis* derived cell line (KC cells).

To date, more than 36 distinct serotypes of BTV have been isolated from ruminants including sheep, cattle and goats [3]. The BTV genome is composed of ten segments of double stranded RNA. BTV serotype is determined by the virus outer capsid and cell attachment protein VP2 (encoded by genome segment 2), which elicits neutralising antibodies in infected vertebrate hosts [4,5,6]. Since 2008, several atypical BTV serotypes have been isolated (primarily from small ruminants, including goats or sheep) that do not replicate either in KC cells, or in adult *C. sonorensis* [3,6,7,8,9]. These viruses are believed to be transmitted by direct contact, from individual infected to uninfected ruminant hosts [10].

Attenuation of BTV vaccine strains, by adapting them to replication in tissue cultures or embryonated chicken eggs, can increase their transplacental transmission in ruminants, often with teratogenic effects that become evident when they are used as vaccines in the field [11,12]. Infection of bovine foetuses was also observed during BT outbreaks in northern Europe caused by field strains of BTV-8 (2006–2010) [11], raising speculation concerning the parental origins of this BTV-8 lineage. Transplacental transmission of a field strain of BTV-2 (passaged once in *Culicoides* (KC) cells and mammalian cells) was demonstrated experimentally in gestating ewes [11]. Vector independent (horizontal) transmission of this BTV-2 was suspected from gestating inoculated to in-contact control ewes. Although the inoculated and control animals were separated by a 0.7 m wide corridor, transmission still occurred between them, indicating horizontal transmission, possibly via an oral route [11]. It is therefore now largely accepted that although the frequency of vector independent transmission of BTV may be strain dependent, it can occur either vertically or horizontally.

Horizontal transmission from infected to non-infected animals has been demonstrated experimentally for BTV-26 and BTV-27 and is now accepted as a primary route of spread for the novel ‘atypical’ bluetongue viruses [9,13,14,15,16,17,18]. Sheep infected with BTV-26 developed minor clinical signs, and although infection in goats was inapparent they did develop viraemia, allowing efficient horizontal transmission during unhindered contact with uninfected animals. BTV-27 did not cause detectable viraemia in sheep or cattle, although infected goats developed minor clinical signs and viraemia, allowing the virus to be transmitted horizontally to other in-contact goats [15].

The segmented nature of the BTV genome allows high frequency reassortment (exchange of genome segments) when two or more distinct viruses infect the same cells of either vertebrate hosts or midge vectors [19]. A reassortant virus, isolated as a contaminant of a sheep pox vaccine grown in ovine testis cells, was shown to contain individual genome segments derived from both vectored and atypical BTVs. This virus has been characterized as BTV-28 [20]. Like BTV-26 from Kuwait [18], the reassortant BTV-28 failed to replicate in *Culicoides* derived KC cells, suggesting that reassortment had occurred during infection of mammalian cells. Unlike BTV-26 or -27, sheep experimentally infected with BTV-28, as well as in-contact but non-inoculated sheep, developed viraemia and severe clinical signs of bluetongue that lasted for several weeks [16]. These findings highlight the potential importance of reassortment between atypical BTVs (that may cause less severe clinical signs [9,10,14,15,21]) and vectored BTVs that can cause severe disease. This could lead to the generation and emergence of novel field strains that are virulent but can be transmitted during periods when adult *Culicoides* vectors are absent, potentially extending both the seasonal and geographic distribution of bluetongue outbreaks [5,6].

Using reverse genetics, we have generated a BTV-1 that induces only relatively mild clinical signs in IFNAR^(−/−)^ mice. This BTV-1 replicates efficiently in mammalian or *Culicoides* derived cells. Reassortants that were generated by insertion of individual BTV-26 genome segments into the genome/genetic backbone of this BTV-1, were used to infect IFNAR^(−/−)^ mice, to identify those genome segments of BTV-26 that influence or support horizontal transmission.

## 2. Materials and Methods

### 2.1. Wild Type Viruses, Cell Cultures and Virus Titrations

BSR baby hamster kidney cells [22] were grown at 37 °C in Dulbecco’s modified Eagle medium (DMEM) supplemented with 10% foetal bovine serum and 100 IU of penicillin/100 µg of streptomycin per mL, under 5% CO_2_.

Wild type BTV-1(RSArrrr/01) and BVT-26(KUW2010/02) were obtained from the dsRNA virus collection at the Pirbright Institute (www.reoviridae.org/dsRNA_virus_pro-teins/ReoID/BTV-isolates.htm, accessed on 2 November 2021). BSR cells were infected with the viruses at a multiplicity of infection (MOI) of 0.1 PFU/cell until full cell lysis was observed (72 h post-infection). The viruses were grown once in BSR cells, then titrated in BSR cells by a plaque assay, as previously described [4,23].

### 2.2. Extraction and Analysis of RNA

Infected BSR cells showing full cytopathic effect (CPE), were pelleted by centrifugation at 2000× *g* for 10 min at 4 °C. The pellet was dissolved in 1 mL of TRIzol by vigorous shaking, followed by addition of 200 µL of chloroform and further shaking for 1 min. For RNA extraction from mouse blood, 10 µL of blood were dissolved in 1 mL of TRIzol by vigorous shaking [4]. Tubes were left on ice for 30 min prior to centrifugation at 12,000× *g* for 10 min at 4 °C. The supernatant was collected and mixed vigorously with an equal volume of isopropanol, incubated at −20 °C for 1 h and centrifuged at 18,000× *g* for 10 min. The supernatant was discarded, and the pellet washed with 75% ethanol. Excess ethanol was removed, and the pellet left to dry. The pellet was dissolved in 50 µL of nuclease-free water.

RNA extracted from BSR cells was subjected to further purification by precipitation in 2 M (final concentration) lithium chloride over night at 8 °C as previously described [24]. Lithium chloride precipitated dsRNA was dissolved in 100 µL of RNase-free water and treated with an equal volume of phenol/chloroform/isoamyl alcohol (25:24:1, Sigma, St. Louis, MO, USA) before being analysed by polyacrylamide gel electrophoresis (PAGE).

### 2.3. PAGE Analysis

PAGE analysis was performed as previously described [25]. Briefly, a 7.5% polyacrylamide gel was prepared in tris/glycine buffer. A dsRNA solution (250 ng/µL) was mixed with an equal volume of Laemmli’s 2× buffer [26]. The gel was run at 25 mA for 1 h and 45 min, followed by staining in ethidium bromide.

### 2.4. Preparation of Full-Length cDNAs from dsRNA

Full-length cDNAs were prepared using the FLAC method [27]. Briefly, an anchor primer (GACCTCTGAGGATTCTAAAC/*iSp9*/TCCAGTTTAGAATCC-OH3′) was ligated to the 3′ ends of the dsRNA. Ligations were carried out in a volume of 20 µL containing 10 µL of total RNA extract, 1 µg (in 1 µL) of anchor primer and 10 units of RNA ligase (New England Bio Labs, Ipswich, MA, USA). The reaction was incubated at 10 °C overnight. Excess non-ligated anchor primer was removed by electrophoresis on 1% agarose gel in TAE buffer. The total dsRNA was recovered from the gel in a volume of 15 µL using RNaid kit, as previously described [24].

Prior to reverse transcription (RT), the dsRNA was heat denatured at 99 °C for 3 min. The RT reaction contained 11 µL of heat denatured dsRNA (1 µg), 2 µL of 10 mM dNTP mix and 4 µL of AMV RT buffer and 1 µL (21 units) of AMV reverse transcriptase (Roche). The reaction was incubated at 37 °C for 1 h, allowing the anchor primer to self-prime, generating a full-length cDNA copies of each dsRNA segment, which were used for PCR.

Full-length cDNAs were PCR amplified using KOD Hot Start DNA Polymerase as described by the manufacturer in presence of primer 5-15-1(5′-GAGGGATCCAGTTTAGAATCCTCAGAGGTC-3′).

### 2.5. Reverse Engineering of BTV-1

Forward and reverse primers were designed from the sequences of BTV-1RSArrrr/01 for each of the 10 genome segments. All forward primers included the minimal T7 promoter sequence at their 5′ end, (TAATACGACTCACTATA) immediately preceding the viral 5′ termini. Forward and reverse primers (Table S1) were designed to amplify each full-length genome segment of BTV-1RSArrrr/01. PCR amplifications were performed using KOD Hot Start DNA Polymerase, as described by the manufacturer, in presence of primers designed for each genome segment.

PCR products were analysed by agarose gel electrophoresis and purified using Qiagen PCR purification columns as described by the manufacturer. Capped mRNAs were generated from linear PCR products by in-vitro transcription using the mMessage mMachine T7 Ultra Kit (Life Technologies, Carlsbad, CA, USA) as previously described [18] and purified using the MEGAclear transcription cleanup kit (Ambion, Austin, TX, USA). Figure S1 shows a schematic representation of the PCR amplification and transcription reaction using the PCR products.

BSR cells grown to 80–90% confluence in 6 well plates were lipofected (lipofectamine 2000) with a mixture consisting of 500 ng from each mRNA transcripts corresponding to Seg-1, Seg-2, Seg-3, Seg-4, Seg-5, Seg-8 and Seg-9 of BTV-1. At 16 h post-lipofection, a second set of transcripts (500 ng each) corresponding to all 10 genome segments of BTV-1 were again lipofected into the same wells. The cells were incubated at 37 °C for up to 5 days and buffered if necessary, using 7.5% NaHCO_3_ to avoid acidification of the culture medium. On day 5 cells were scraped from the wells, passaged into T25 cell culture flasks, incubated at 37 °C and monitored for CPE. On day 5 cells were scraped and centrifuged. The resulting cell pellets were resuspended in calcium and magnesium-free PBS, and subjected to 10 strokes of Dounce homogenisation, before treating with an equal volume of Vertrel-XF, as previously described [28]. The supernatant was used for plaque purification (twice), as previously described [4,23]. Plaques were collected and the virus amplified once in BSR cells before being assessed in IFNAR^(−/−)^ mice. A clone (BTV-1RG_C7_) which induced milder clinical signs in IFNAR^(−/−)^ mice was selected for reverse genetic studies to generate reassortants of BTV-26 in the BTV-1 backbone.

### 2.6. Reverse Engineering of Reassortants Containing BTV-26 Genome Segments in the BTV-1 Genetic Backbone

Genome segments of the selected BTV-1 clone were converted into cDNA using FLAC as described above. Forward and reverse primers containing enzyme restriction sites were used to facilitate cloning of cDNA copies of each BTV-1 genome segment into plasmids, as shown in Table 1. All forward primers included the minimal T7 promoter sequence at their 5′ end, (TAATACGACTCACTATA) immediately preceding the viral 5′ termini. Plasmids and PCR products, digested with appropriate restriction enzymes (as described in Table 1), were ligated using T4 DNA ligase and used to transform chemically competent Stbl2™ (Invitrogen, Waltham, MA, USA) bacteria. Plasmid clones were purified from colonies grown in liquid cultures using the Qiaprep miniprep kit and commercially sequenced using the BigDye Terminator v3.1 Cycle Sequencing Kit (Invitrogen).

Plasmids were linearised using the appropriate restriction enzyme (Table 1), then purified using Qiaquick purification kit and used in transcription reactions to generate full length T7 transcripts. The primers used to generate full-length linear PCR products corresponding to each of the 10 individual genome segments of BTV-26 are shown in Table 2. All forward primers included the minimal T7 promoter sequence at their 5′ end, (TAATACGACTCACTATA) immediately preceding the viral 5′ termini. PCR amplifications were performed using KOD Hot Start DNA Polymerase as described by the manufacturer, in the presence of primers designed for each genome segment. PCR products were analysed by agarose gel electrophoresis and purified using Qiagen PCR purification columns, as described by the manufacturer.

Capped mRNAs were generated from PCR products and purified as described above. A schematic representation of cloning the full-length cDNAs of BTV-1 and T7 transcription is shown in Figure S2. Reassortants were generated by lipofecting monolayers of BSR cells grown in 6 well plates as described above, using a mixture of mRNA transcripts of BTV-1 and BTV-26 in the desired combination (Figure S3). Reassortants were collected, treated with Vertrel XF, plaque purified once on BSR cells and propagated once in BSR cells. This passage was either stored at -80°C or used to extract dsRNA for PAGE analysis.

### 2.7. IFNAR^(−/−)^ Mice

IFNAR^(−/−)^ mice (genetic background: A129SvEvBrd) [29] were a gift from Professor Michel Aguet (ISREC, Ecole Polytechnique Fédérale de Lausanne, Switzerland). IFNAR^(−/−)^ mice can be lethally infected with orbiviruses, including BTV (serotypes 1 to 24). These mice have been used in several previous studies [4,30,31] and are regarded as a useful small animal model to study the replication and vaccinology of BTV. 

Six to fourteen weeks old female IFNAR^(−/−)^ mice were used throughout the experiments. The mice were held in plastic cages with wood-chip bedding, fed ad libitum and kept in standardised conditions (21 °C, 12:12 light/dark cycle) at an SPF animal facility. 

### 2.8. Comparison of Phenotypes of BTV-1RG and Wild Type BTV-1RSArrrr/01 in IFNAR^(−/−)^ Mice

Groups, of 4 mice, were used to assess the replication and clinical signs caused by infection with wild type BTV-1RSArrrr/01, or genetically engineered BTV-1RG_C7_. Mice in each group were inoculated intraperitoneally with 10^2^ PFU/mouse of wild type BTV-1RSArrrr/01 or BTV-1RG (Figure S4). Blood samples (10–30 µL) were collected on day 0, 4, 8 and 11 post-infection (pi). RNA was extracted using TRIzol as described by the manufacturer. Viraemia was assessed by real-time RT-PCR of RNA extracted from blood. Real-time RT-PCR for BTV was performed using primers and probes targeting genome segment 10 (BTV_S10_F: TGGAYAAAGCRATGTCAAA, BTV_S10_P: FAM-ARGCTGCATTCGCATCGTACGC-BHQ1, BTV_S10_R: ACRTCATCACGAAAC-GCTTC) [32].

### 2.9. Infection of IFNAR^(−/−)^ with Reverse Engineered Reassortant Viruses and Assessment of Horizontal Transmission Potential

Two groups of 4 female mice (6–8 weeks old) were used to assess the potential for horizontal transmission (HT) of BTV-1RG_C7_ or BTV-26 (Figure S4). In each group, two mice were inoculated intraperitoneally, either with 10^2^ PFU of BTV-1RG_C7_ or BTV-26/mouse then kept in a cage in direct contact with 2 non-inoculated mice. Blood samples (10–30 µL/mouse) were collected on days 0, 4, 8 and 11. The potential for HT of each reassortant virus was also assessed using the same settings. Experiments assessing HT potential were repeated twice. The HT potential of each of the 10 reassortant viruses was also evaluated in a similar manner. This experiment was repeated once with older animals (10–14 weeks old). Animals were euthanized on day 15 post-inoculation. The WT BTV-1RSArrrr/01 caused clinical signs in mice, ultimately killing them by day 5–6 pi. This would significantly reduce the time that uninoculated mice could be exposed ‘in contact’ to the infected mice. In contrast, BTV-1RG_C7_ caused only minor signs of infection, allowing contact with uninfected mice for the duration of the experiment (up to 15 days) providing more opportunity for horizontal transmission. The BTV-1RG_C7_ backbone was therefore used to reverse engineer reassortants of BTV-26.

## 3. Results

### 3.1. Rescue of BTV1-RG and Plaque Purification of Clones

**Figure 1:**
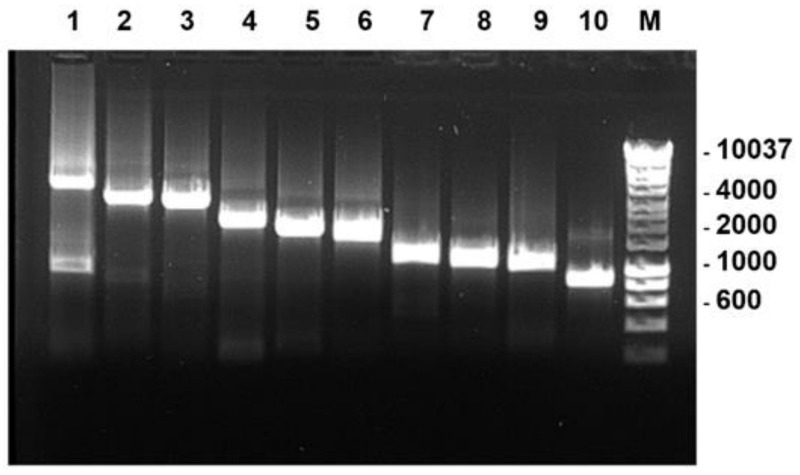
T7 full-length PCR products corresponding to genome segments 1–10 of BTV-1 (lanes 1–10) analysed by 1% agarose gel electrophoresis. These PCR products were used for in vitro transcription with T7 polymerase. Lane M: DNA size marker labelled in base pairs (bp).

Full-length PCR products of the individual genome segments of BTV-1RSArrrr/01 were successfully generated using the FLAC method (Figure 1).

**Figure 2:**
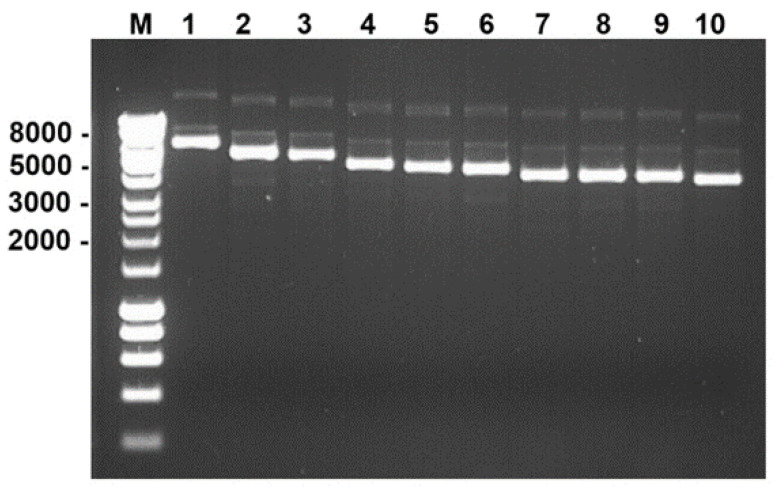
Agarose gel electrophoresis analysis of the pGEX-4T-2 plasmid clones containing the Figure S1. of BTV-1RG_C7_ (lanes 1–10). Lane M: DNA size marker labelled in bp.

T7 transcripts generated with the mMessage mMachine T7 Ultra Kit were transfected into BSR cells, resulting in the rescue of BTV-1 by reverse genetics. Vertrel XF was used to release virus particles from cell debris [28,33,34]. Following Vertrel treatment and plaque purification, ten clones were collected, and plaque purified twice in BSR cells. These clones were amplified once in BSR cells and used to infect IFNAR^(−/−)^ mice. Nine clones induced typical signs of infection in inoculated mice, which died by day 6 pi. However, clone 7, which was designated BTV-1RG_C7_, induced only minor clinical signs and was used for further reverse genetic studies. The dsRNA from this clone was converted into cDNA, PCR amplified and cloned by restriction digestion/ligation into pGEX-4T-2 plasmid. Plasmid clones corresponding to the 10 genome segments of BTV-1RG_C7_ were purified by miniprep (Figure 2).

### 3.2. The Full-Length Sequence of BTV-1RG_C7_ Genome

The plasmid clones which served as templates for linear PCR products used to generate T7 transcripts, were sequenced using the BigDye Terminator v3.1 Cycle Sequencing Kit. Nucleotide (Nt) and/or amino acid (AA) substitutions were identified in genome segments 1, 2, 3, 4, 5, 6 and 8 of BTV-1RG_C7_, compared to the consensus reference sequence of the wild type BTV-1 [BTV-1RSArrrr/01] (Table 3). No substitutions were detected in segments 7, 9 or 10. As shown in Table 3, most of the depicted nucleotide substitutions are synonymous and thus do not incur changes in the amino acid sequences. However, some nucleotide substitutions incurred amino acid changes including radical or conservative substitutions in the VP2, VP3, VP5 and NS1 (Table 3) as defined by the amino acid exchangeability matrices [35]. There were no insertions or deletions in any of the sequences. 

The plaque purified clone 3 of BTV-1RG (BTV-1RG_C3_, a clone with a virulent phenotype) was sequenced in order to attempt identifying specific residues which might contribute to the attenuated phenotype of BTV-1RG_C7_. The sequence of the full-length genome of BTV-1RG_C3_, was almost identical to that of the parental BTV-1RSArrrr/01. Only two synonymous nucleotide substitutions were identified in genome segment 1 (G3113A) and 6 (A310T) of this clone, and these two substitutions were also detected in BTV-1RG_C7_. This analysis does not allow us to clearly identify which nucleotide and/or amino acid changes contribute to the attenuated phenotype of BTV-1RG_C7_. Additional work will be necessary to reverse engineer the various depicted substitutions into the original sequence of BTV-1RSArrr/01. 

### 3.3. Generation of Reassortants

**Figure 3:**
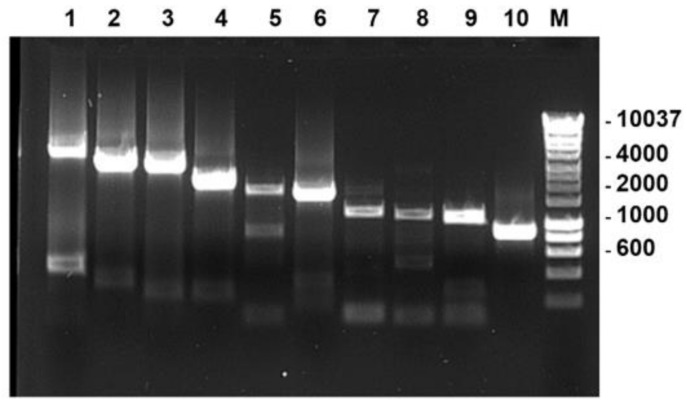
T7 full-length PCR products corresponding to genome segments 1–10 of BTV-26 (lanes 1–10), used for in-vitro transcription with T7 polymerase. The transcripts were used to generate reassortants of BTV-26 in the BTV-1RG_C7_ backbone. Lane M: DNA size marker labelled in bp.

Linearised plasmid clones of BTV-1RG_C7_ were obtained by digesting the plasmids with BsmBI or BsaI enzymes (Table 1). Full-length cDNAs of BTV-26, prepared using FLAC, were PCR amplified with the primers described in Table 2. Linearised plasmids and full-length PCR products (shown in Figure 3) were used for synthesis of T7 RNA transcripts of the BTV-1 or BTV-26 genomes segments. All reassortants were generated by lipofecting T7 transcripts into BSR cells according to the combinations shown in Figure S3.

**Figure 4:**
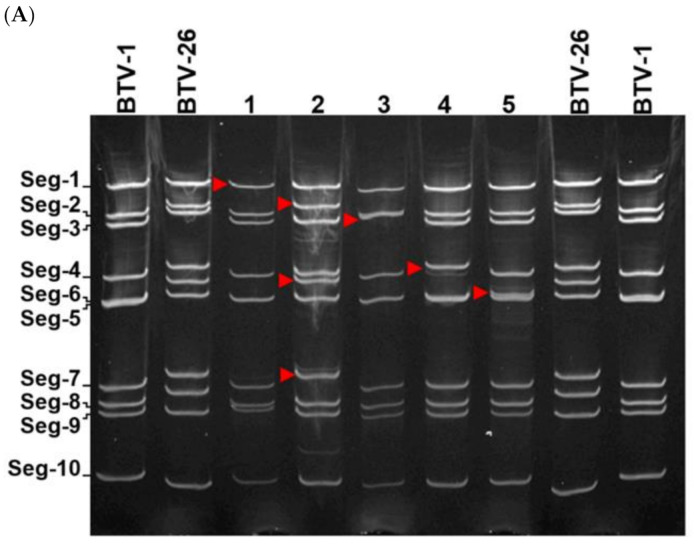

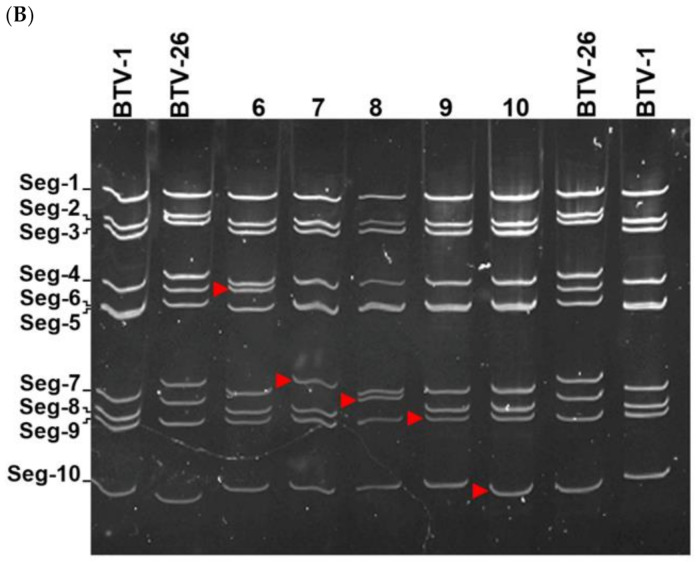
Analysis of the electropherotypes of the 10 reassortants of BTV-26 in BTV-1 backbone in 7.5% polyacrylamide gel. Segments derived from BTV-26 are indicated by a red arrow. (**A**) shows the electropherotypes of BTV-1RG_C7_ and BTV-26, as well as RGBTV1:1_(26)_ in lane 1, RGBTV1:2,6,7_(26)_ in lane 2RGBTV1:3_(26)_ in lane 3, RGBTV1:4_(26)_ in lane 4 and RGBTV1:5_(26)_ in lane 5. (**B**) shows the electropherotypes of BTV-1RG_C7_ and BTV-26, as well as: RGBTV1:6_(26)_ in lane 6, RGBTV1:7_(26)_ in lane 7, RGBTV1:8_(26)_ in lane 8, RGBTV1:9_(26)_ in lane 9 and RGBTV1:10_(26)_ in lane 10.

Reassortants were named RGBTV1:1_(26)_, RGBTV1:2,6,7_(26)_, RGBTV1:3_(26)_, RGBTV1:4_(26)_, RGBTV1:5_(26)_, RGBTV1:6_(26)_, RGBTV1:7_(26)_, RGBTV1:8_(26)_, RGBTV1:9_(26)_, RGBTV1:10_(26)_. The triple reassortant RGBTV1:2,6,7_(26)_, (containing genome segments 2, 6 and 7 derived from BTV-26) was generated based on previously reported data, where rescue of a BTV-26 Seg-2 mono-reassortant in the BTV-1 backbone was unsuccessful [18]. Reassortants were plaque purified in BSR cells and further expanded by passaging once in BSR cells. Extracted and purified dsRNA was analysed by 7.5% PAGE (Figure 4). Migration patterns of the dsRNA genome segments were compared and found to match the same segments in the parental virus (Figure 4). All reassortants of BTV-26 in the BTV-1RG_C7_ backbone replicated efficiently in BSR cells as shown from the levels of dsRNA in Figure 4, producing very similar titers by plaque assay (about 10^8^ PFU/mL of tissue culture lysates). Reassortants RGBTV1:1_(26)_, RGBTV1:2,6,7_(26)_, RGBTV1:3_(26)_ do not replicate in KC *Culicoides* cells, or as reported by Pullinger et al. [18], in adult *Culicoides*, while the other 7 reassortants all replicate efficiently in KC cells. 

### 3.4. Replication of BTV-1RSA and BTV-1RG_C7_ in IFNAR^(−/−)^ Mice

**Figure 5:**
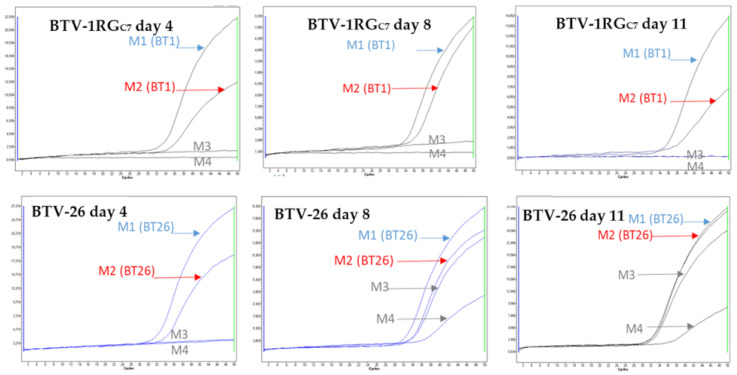
Real-time RT-PCR amplification curves of RNA extracts from blood samples of animals inoculated with BTV-1RG_C7_ or BTV-26 (M1 and M2 are the mice inoculated with the virus. M3 and M4 are the in-contact mice).

Groups of 4 IFNAR^(−/−)^ mice were inoculated with BTV-1 wild type (WT) or BTV-1RG_C7_. Blood samples collected from inoculated and non-inoculated animals on days 0, 4, 6 and 8 were tested by real-time RT-PCR. The WT BTV-1RSArrrr/01 caused clinical signs in mice, ultimately killing them by day 5–6 pi. Blood samples collected on day 4 pi had Ct values of 22–25. In contrast, BTV-1RG_C7_ caused only minor signs of infection (lacrimation on day 4–5 post inoculation and scruffy fur) and blood samples collected on day 4 pi had Ct values of 32–35 and all of the mice recovered by day 6–7. The nine other clones of BTV-1RG replicated in IFNAR^(−/−)^ mice all generating severe clinical signs, with Ct values of 25–30 on day 4 pi, and all mice dying by day 5–6 pi. Non-inoculated control mice showed no clinical signs of infection, and their blood samples were all negative for BTV RNA, as observed by real-time RT-PCR (Figure 5). The mechanism responsible for reduced virulence of BTV-1RG_C7_ has not yet been determined. 

### 3.5. Horizontal Transmission of BTV-26

In horizontal transmission experiments, the two mice inoculated with BTV-26 showed minor clinical signs of infection with lacrimation on day 4–5 but had recovered by day 6–7. The non-inoculated in-contact mice also showed minor signs of lacrimation on day 8–9 but had recovered by day 11–12. On day 4, both inoculated mice were positive for BTV RNA (Ct values ~32–36). On day 8, both non-inoculated, in-contact mice were also positive for BTV RNA (Ct values of ~36–38) and they remained positive when retested on day 11 (Figure 5), demonstrating horizontal transmission of BTV-26. The non-inoculated mice in HT experiments with BTV-1RG_C7_ did not become infected (Figure 5). This set of experimental results confirms the usefulness of IFNAR^(−/−)^ mice as a model to study horizontal transmission of BTV-26.

### 3.6. Identification of the BTV-26 Genome Segments That Support Horizontal Transmission

**Figure 6:**
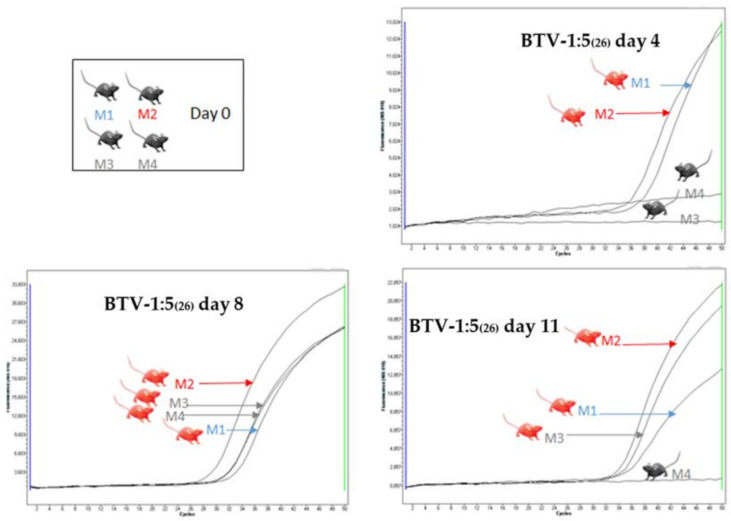
Real-time RT-PCR amplification curves for RNA extracts from blood samples of mice inoculated with BTV-1:5_(26)_. On day 4 both inoculated mice (M1 and M2) are positive by real-time RT-PCR. On day 8 and 11, both in-contact mice (M3 and M4) were positive for BTV RNA. In one of the experiments, one in-contact mouse (M4) became negative on day 11 of the experiment.

**Figure 7:**
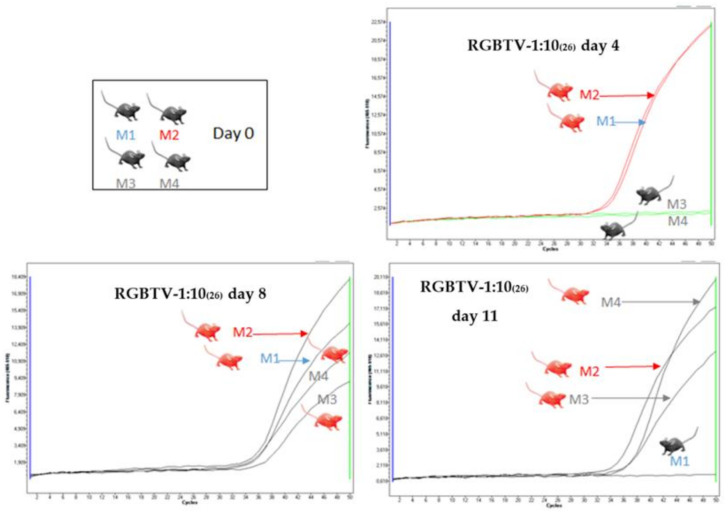
Real-time RT-PCR amplification curves of RNA extracts from blood samples of mice inoculated with BTV-1:10_(26)_. On day 4 both inoculated mice (M1 and M2) were positive by real-time RT-PCR. On day 8 and 11 both in-contact mice (M3 and M4) were positive for BTV RNA. In one of the experiments, one mouse (M1) which was inoculated on day 0, became negative on day 11 of the experiment.

Minor clinical signs (transient lacrimation and scruffy fur) were observed on days 4–5 during HT experiments, in mice inoculated with 10^2^ PFU of the BTV-26/ BTV-1RG_C7_ reassortants. All of the reassortants replicated in the inoculated mice (Table 4), with Ct values on day 4 ranging from 22 to 37, and all of the mice had recovered by day 11–12. Clinical signs consistent with horizontal transmission were also observed in the non-inoculated mice in the group receiving reassortants RGBTV1:5_(26),_ or RGBTV1:10_(26)._ For RGBTV1:5_(26)_, both in-contact mice showed, evidence of viraemia on day 8, while one of these two mice tested negative on day 11 (Figure 6). For RGBTV1:10_(26)_ (NS3/NS5 of BTV-26), both in-contact mice were positive on day 8 and were retested positive on day 11 of the experiment (Figure 7).

**Figure 8:**
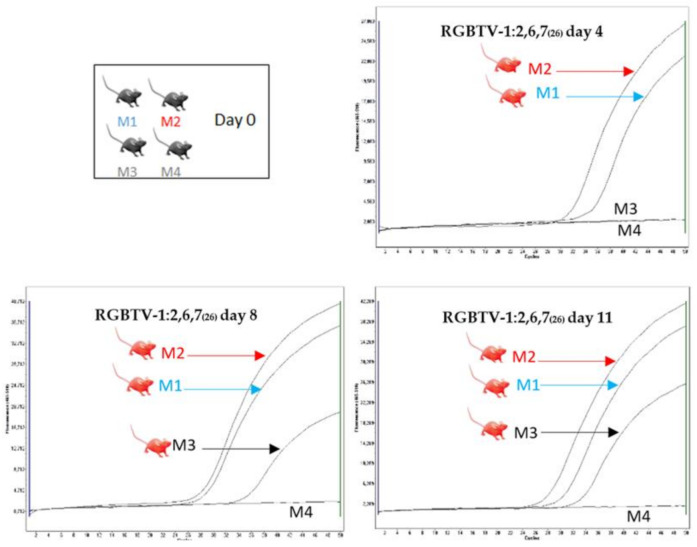
Real-time RT-PCR amplification curves of RNA extracts from blood samples of mice inoculated with BTV-1:2,6,7_(26)_. On day 4 both inoculated mice were positive by real-time RT-PCR. On day 8 and 11 one in-contact mouse was positive for BTV RNA.

These results indicate that Seg-5/NS1 or Seg10/NS3-NS5 of BTV-26 are both individually sufficient to permit horizontal transmission of these reassortants strains and are therefore likely to influence horizontal transmission of BTV-26, at least in the mouse model. On one occasion, one in-contact mouse was also positive (minor clinical signs and real-time RT-PCR) in the group inoculated with reassortant RGBTV1:2,6,7_(26)_ (expressing VP2, VP5 and VP7 of BTV-26) (Figure 8), indicating that one or more, or possibly this combination of three BTV-26 genome segments may also play some role in supporting HT of these reassortants.

## 4. Discussion

BTV is an arbovirus that is usually transmitted between individual susceptible hosts by competent species of *Culicoides* biting midges [1,6,36]. Horizontal transmission of vectored BTVs has been linked to contaminated semen from infected animals. Vertical transmission of BTV was first described with attenuated vaccine strains belonging to serotypes 10, 11, 13 and 17 [37]. After BTV-8 emerged in northern-Europe in 2006, it was rapidly observed to cross the placental barrier [38,39,40,41]. However, experimental infection studies with gestating animals that were intended to confirm vertical transmission also revealed potential for the direct transmission of other specific conventional BTV serotypes. For instance, direct transmission of BTV-2 was observed from inoculated to non-inoculated control gestating ewes [11]. The northern-European strain of BTV-8 was shown to infect bovine foetuses in utero and infection of heifers also occurred after ingestion of contaminated tissues, such as placenta [38,42], confirming horizontal transmission by an oral route.

BTV can infect wild carnivores preying on meat/organs of infected animals in Africa [43] and lynx that were fed meat contaminated with the northern-European strain of BTV-8, became infected and died [44]. It has been suggested that this finding may be linked to the use of live attenuated vaccines in the field. This hypothesis is supported by other findings, where vaccination of gestating dogs with a BTV-11vac contaminated multivalent modified live canine distemper virus, adenovirus and parvovirus vaccine lead to abortion [45]. The general hypothesis that transplacental transmission of BTV is linked to live attenuated vaccine strains is now largely accepted [12], although historically these vaccines were widely used and persisted in the field, suggesting that this may now be a more generalized characteristic of BTV field strains.

Horizontal transmission of the northern-European BTV-8, or the South-African BTV-1 vaccine strain from infected to in-contact gestating ewes, could have occurred by ingestion of saliva/blood contaminated feed or water [39,46]. However, in a recent study, UK and French isolates of BTV-8 (UKG2007 and FRA2017 respectively) did not transmit horizontally from infected to non-infected in-contact ewes (non-gestating) [47]. Other studies also showed that strains of BTV-4 do not transmit horizontally in sheep, goat or calves [48].

BTV-25 is the first discovered atypical BTV serotype but was not able to replicate in either mammalian or insect cell cultures and may therefore be different to the other novel BTV serotypes. BTV-25 was not detected in milk, urine, nasal or ocular secretion, or in the faeces of infected goats [49]. These observations suggest that the ‘atypical’ BTVs can spread efficiently by direct contact, in contrast with the low efficiency contact transmission of conventional vectored BTVs.

Previous studies which reported possible or potential direct transmission of vectored BTVs in ruminants were all conducted with gestating animals, which showed clinical signs of infection. However, the horizontally transmitted BTV-25 caused only limited clinical signs and was not found in biological fluids, including milk, urine, nasal and ocular secretion, or in faeces of infected goats, suggesting that the opportunities for the virus to contaminate animal feed or water would be very limited [49].

Even in the absence of clinical signs, direct contact transmission of BTV-26 or BTV-27 from experimentally infected to in-contact goats, is very efficient [14,15]. It is therefore important to understand if the state of gestation exerts an influence on the way that vectored BTV serotypes (or at least those which can be transmitted horizontally) could be secreted/excreted. The possibility that in-utero infection of the foetus leads to losses/escape of intrauterine fluids contaminated with the virus, promoting oral contamination of in-contact animals needs further clarification.

The loss of transmissibility of atypical BTV serotypes by *Culicoides* vectors [17,18] has been linked to 3 genome segments, each encoding one structural protein: (i) Seg-1 encoding the viral RNA dependent RNA polymerase, VP1, (ii) Seg-2 encoding outer-capsid protein VP2 that binds to cell surface receptors and (iii) Seg-3 encoding the innermost sub-core shell protein VP3(T2) of the virus particle, which is in direct contact with the dsRNA genome. The outer-core VP7 protein (encoded by Seg-7) seemed to play a lesser role in reducing replication of BTV-26 when a mono-reassortant of BTV-26 Seg-7 in a BTV-1 backbone, was used to infect *Culicoides* cells [18].

Our reverse genetics studies led to isolation of a BTV-1 clone (designated BTV-1RG_C7_), derived from the BTV1-RSArrrr/01, which caused less severe clinical signs of infection in IFNAR^(−/−)^ mice, ultimately with a full recovery. Sequence analysis showed several nucleotide substitutions in genome segments 1, 2, 3, 4, 5, 6 and 8 of BTV-1RG_C7_, some of which resulted in amino acid changes. It is therefore likely that the attenuated phenotype of clone BTV-1RG_C7_ is linked to one or more of these mutations.

In the current study, our assessment of genome segments which influence or support horizontal transmission of an atypical BTV, strongly suggests that genome segments 2, 5 and 10 of BTV-26 can influence vector independent direct horizontal transmission in a mouse model. These genome segments encode outer capsid protein VP2 (Seg-2), non-structural protein NS1 (Seg-5) and non-structural proteins NS3 and NS5 (Seg-10). VP2 of vectored BTV binds efficiently to insect cell surface while VP2 of non-vectored BTVs fails to do so [17]. VP2 of both groups is functional in mammalian cell cultures. The results of our study suggest that segment 2 (encoding VP2) of a non-vectored BTV may also exert some influence on horizontal, in-contact transmission. However, cellular receptors for BTV VP2 may differ in terms of binding capacity and affinity in the different vertebrate species.

Studies conducted with other viruses including SARS-CoV-2 have shown that lentiviruses pseudo-typed with the variants of the S2 spike protein had different capacity to interact with the well characterised SARS-CoV-2 ACE2 receptor (Angiotensin Converting Enzyme 2: ACE2) of a range of animals. ACE2 of mice came out as the one with poor binding properties [50]. BTV receptors are not fully characterised, and BTV may use several different entry mechanisms for structurally different virus particles, and the two different serotypes that have been studied appear to use different cell entry mechanisms [51,52] although the glycophorin of sheep red blood cells appear to bind the virus with high affinity [53]. There are inherent differences between the VP2 of vectored and non-vectored BTVs in term of their sequences [9]. Identification of specific receptors which bind the outer-capsid protein VP2 of vector independent horizontally transmitted BTVs may be important to fully understand changes to the route of transmission used by atypical BTV serotypes.

As observed with other viruses [54,55], adaptation to specific receptors, could provide an evolutionary advantage for the atypical BTV serotypes (which can no longer replicate in the insect vector), allowing them to directly infect their hosts. Other members of the family *Reoviridae* including the orthoreoviruses and rotaviruses also infect mammals, via oral-fecal and/or respiratory routes, exhibiting tissue tropism and specificities for different receptors in a strain/serotype dependent manner [56,57]. For instance, Rotaviruses use histo-blood group antigens as receptors, but several other receptors have been proposed for rotavirus cell-entry including heat shock protein 70, integrins, tight junction protein ZO-1, junction adhesion molecule A and occludin [58,59,60,61,62,63,64].

The role of BTV genome segments 5 and 10 in supporting horizontal transmission could relate to the role played by these proteins in cell exit [65], which may determine the availability of virus particles for transmission to other hosts. When NS1 is abundant, the virus leaves the cells by lysis as it is the case usually in mammalian cells during advanced stages of infection. When NS3 is abundant, it favours release of the virus from insect cells [66,67,68]. The virus can also leave the cell in secretory endosomes [69]. Although NS1 is a non-structural protein, earlier studies showed that it is present on the temporary envelope of viral particles leaving the cells [65]. Beyond cell exit, NS1 plays the role of a positive regulator of viral protein synthesis in infected cells. In addition to its role in cell exit [65], NS3 is involved in interferon antagonism [70].

It is noteworthy that BTV-28, which is pathogenic to sheep [16,20], is a reassortant between vectored and atypical bluetongue viruses. BTV-28 does not grow in *Culicoides* cells and can be transmitted directly from infected to non-infected sheep. It has all three genome segments 2, 5 and 10 related to those of BTV-26. It is therefore possible that these three genome segments of atypical BTV serotypes collectively play a role in entry and exit of tissues which vectored BTVs do not traditionally infect (or infect with lower efficiency), thus facilitating vector independent direct horizontal transmission of an orbivirus.

## 5. Conclusions

This study indicates that genome segments 5 and 10 can individually support the horizontal, in-contact transmission of an atypical bluetongue virus (BTV-26) in a mouse model. These are different genome segments to those previously identified as restricting *Culicoides* infection by BTV-26 (segments 1, 2 and 3, with a lesser effect by segment 7 [18]). There is also some indication that genome segment 2 might exert some influence over horizontal transmission of BTV-26 in mice. Studies in the natural hosts of BTV will be necessary to determine whether these observations are also true in ruminants.

## Figures and Tables

**Table 1:** Primers designed for PCR amplification of full-length cDNAs of genome segments 1–10 of BTV-1, for cloning into the pGEX-4T-2 plasmid. The minimal T7 promoter in the forward primer is shown in bold. Restriction enzyme used to facilitate cloning into the pGEX-4T-2 plasmid are underlined. Enzyme used to linearise the plasmid before T7 transcription is shown in the column to right of the table.

Virus/Segment	Primer Sequence 5′→3′	Cloning into pGEX	Linearisation
BTV1-1T7BamF	tctagcGGATCC**TAATACGACTCACTATA**GTTAAAATGCAATGGTCGCAATC	BamHI	
BTV1-1BsmBI-NotR	tacagtaaGCGGCCGCGTCTCAGTAAGTGTAATGCGGCGCGTGC	NotI	BsmBI
BTV1-2T7EcoF	tctagcGAATTC**TAATACGACTCACTATA**GTTAAAATAGTAGCGCGATGGATG	EcoRI	
BTV1-2BsmBI-NotR	tacagtaaGCGGCCGCGTCTCAGTAAGTCTAATAGTGCGCGGATC	NotI	BsmBI
BTV1-3T7EcoF	tctagcGAATTC**TAATACGACTCACTATA**GTTAAATTTCCGTAGCCATGGCTG	EcoRI	
BTV1-3BsmBI-NotR	tacagtaaGCGGCCGCGTCTCAGTAAGTGTGTTCCCGCTGCCGC	NotI	BsmBI
BTV1-4T7EcoF	tctagcGAATTC**TAATACGACTCACTATA**GTTAAAACATGCCTGAGCCACACG	EcoRI	
BTV1-4BsaI-NotR	tacagtaaGCGGCCGCGGTCTCAGTAAGTTGTACATGCCCCCCTC	NotI	BsaI
BTV1-5T7EcoF	tctagcGAATTC**TAATACGACTCACTATA**GTTAAAAAAGTTCTCTAGTTGGC	EcoRI	
BTV1-5BsmBI-NotR	tacagtaaGCGGCCGCGTCTCAGTAAGTTGAAAAGTTCTAGTAGAGTG	NotI	BsmBI
BTV1-6T7EcoF	tctagcGAATTC**TAATACGACTCACTATA**GTTAAAAAGTGCGCCCTTAGCGAA	EcoRI	
BTV1-6BsaI-NotR	tacagtaaGCGGCCGCGGTCTCAGTAAGTGTAAGTGCTTCCCGTCGC	NotI	BsaI
BTV1-7T7EcoF	tctagcGAATTC**TAATACGACTCACTATA**GTTAAAAATCTATAGAGATGGACA	EcoRI	
BTV1-7BsaI-NotR	tacagtaaGCGGCCGCGGTCTCAGTAAGTGTAATCTAAGAGACGTTTG	NotI	BsaI
BTV1-8T7BamF	tctagcGGATCC**TAATACGACTCACTATA**GTTAAAAAATCCTTGAGTCATGGAG	BamHI	
BTV1-8BsmBI-NotR	tacagtaaGCGGCCGCGTCTCAGTAAGTGTAAAATCCCCCCCTAACC	NotI	BsmBI
BTV1-9T7EcoF	tctagcGAATTC**TAATACGACTCACTATA**GTTAAAAAATCGCATATGTCAGCTG	EcoRI	
BTV1-9BsmBI-NotR	tacagtaaGCGGCCGCGTCTCAGTAAGTGTAAAATCGCCCTACGTCA	NotI	BsmBI
BTV1-10T7EcoF	tctagcGGATCC**TAATACGACTCACTATA**GTTAAAAAGTGTCGCTGCCATGCT	EcoRI	
BTV1-10BsmBI-NotR	tacagtaaGCGGCCGCGTCTCAGTAAGTGTGTAGCGCCGCATACCCTC	NotI	BsmBI

**Table 2:** Primers designed for PCR amplification of full-length cDNAs of genome segments 1–10 of BTV-26. The minimal T7 promoter in the forward primer is shown in bold.

Virus/Segment	Primer Sequence 5′→3′
BTV26-S1T7FOR	**TAATACGACTCACTATA**GTTAAAATGCAATGGTCGCGATTAC
BTV26-S1REV	GTAAGTGTAATGCAGCGCGTGT
BTV26-S2T7FOR	**TAATACGACTCACTATA**GTTAAAAGAGCGTTCCACCATG
BTV26-S2REV	GTAAGTGTAAGAGGCCACCG
BTV26-S3T7FOR	**TAATACGACTCACTATA**GTTAAATTTCCGTGGCTATGGC
BTV26-S3REV	GTAAGTGTATTTCCGCTGCTGC
BTV26-S4T7FOR	**TAATACGACTCACTATA**GTTAAAACATGCCTGAGCCAC
BTV26-S4REV	GTAAGTTGTAACATGCCCCCC
BTV26-S5T7FOR	**TAATACGACTCACTATA**GTTAAAAAAGTTCTCTAGTCGGC
BTV26-S5REV	GTAAGTTGAAAAGTTCTATTAGAGTG
BTV26-S6T7FOR	**TAATACGACTCACTATA**GTTAAAAAGTACCCTCTAACTCG
BTV26-S6REV	GTAAGTGTAAGCACCTCCCCC
BTV26-S7T7FOR	**TAATACGACTCACTATA**GTTAAAAATCTATAGAGATGGACACT
BTV26-S7REV	GTAAGTGTAATCTAAGAGACGTA
BTV26-S8T7FOR	**TAATACGACTCACTATA**GTTAAAAAATCCTTAGTCATGGAG
BTV26-S8REV	GTAAGTGTAAAATCCCCCCCT
BTV26-S9T7FOR	**TAATACGACTCACTATA**GTTAAAAAATCGCTTATGTCGGC
BTV26-S9REV	GTAAGTGTAAAACCGCTATATGC
BTV26-S10T7FOR	**TAATACGACTCACTATA**GTTAAAAAGTGTCGCTGCCATG
BTV26-S10REV	GTAAGTGTGTAGTGCCGCATA

**Table 3:** Nucleotide and/or amino acid substitutions detected in the genome of BTV-1RG_C7_ as compared to the reference sequences of BTV-1RSArrrr/01. * Accession numbers of the reference strain of BTV-1RSArrrr/01 used for sequence comparisons.

Segment/Protein	Nucleotide Substitutions	Amino Acid Substitutions	Accession Numbers *
Seg-1/VP1(Pol)	G3113A	Synonymous	JX680457
	G3401A	Synonymous	
Seg-2/VP2 (OC1)	T782C	Synonymous	JX680458
	A924G	N303D (conservative)	
	T2395A	L790Q (radical)	
	G2657A	Synonymous	
Seg-3/VP3 (T2)	G2693T	G893C (radical)	JX680459
Seg-4/VP4(Cap)	A741G	K245E (conservative)	JX680460
	C1091T	Synonymous	
Seg-5/NS1(Tup)	T265C	Synonymous	JX680461/JN848763
	G948A	R305Q (conservative)	
Seg-6/VP5(OC2)	A310T	Synonymous	JX680462
	A312T	Q95L (radical)	
Seg-7/VP7(T13)	None		JX680463
Seg-8/NS2(Vib)	C88T	Synonymous	JX680464
Seg-9/VP6(Hel)/NS4	None		JX680465
Seg-10/NS3/NS5	None		JX680466

**Table 4:** Ct values of the RNA extracted from mice inoculated with the reassortants of BTV-26 in the BTV-1RG_C7_ backbone and from non-inoculated in-contact mice. Ct values ranged from 23 to 37. Positive in-contact mice were identified in the groups inoculated with RGBTV1:2,6,7_(26)_, RGBTV1:5_(26)_ or RGBTV1:10_(26)_.

Reassortant	Inoculated Mice: Ct Range	Non-Inoculated Mice: Ct Range
RGBTV1:1_(26)_	32–37	Negative
RGBTV1:2,6,7_(26)_	26–36	32–34 (1 positive mouse)
RGBTV1:3_(26)_	30–36	Negative
RGBTV1:4_(26)_	26–32	Negative
RGBTV1:5_(26)_	26–30	30–32 (2 positive mice)
RGBTV1: 6_(26)_	22–27	Negative
RGBTV1:7_(26)_	29–33	Negative
RGBTV1:8_(26)_	23–28	Negative
RGBTV1:9_(26)_	26–31	Negative
RGBTV1:10_(26)_	27–30	26–30 (2 positive mice)

## Data Availability

All data are presented in the manuscript.

## Supplementary Materials

The following are available online at https://www.mdpi.com/article/10.3390/v13112208/s1, Figure S1: A schematic representation for generation of transcripts from linear PCR products representing the full-length cDNA copies of BTV genome segments (ARCA: anti-reverse cap analogue), Figure S2: A schematic representation for generating transcripts from restriction enzyme linearised plasmids containing the full-length cDNA copies of BTV genome segments (ARCA: anti-reverse cap analogue), Figure S3: Schematic representation showing RNA combinations used to generate reassortants of BTV-26 in the BTV-1 backbone. The reassortants were designated RGBTV1:X_(26)_ (e.g., RGBTV1:1_(26)_, RGBTV1:5_(26)_), where RGBTV1 refers to the genetic backbone of BTV1-RG_C7_, and X refers to the specific genome segment of BTV-26 introduced into the reassortant, Figure S4: A schematic representation of the horizontal transmission experiment in IFNAR^(−/−)^ mice. Red-coloured mice indicate where replication of a given BTV was detected by real-time RT-PCR, Table S1: Primers designed for PCR amplification of full-length cDNAs of genome segments 1–10 of BTV-1. The minimal T7 promoter in the forward primer is shown in bold.
