# Supplementary material for: Identification of the Genome Segments of Bluetongue Virus Type 26/Type 1 Reassortants Influencing Horizontal Transmission in a Mouse Model
Source: Viruses. 2021 Nov 2;13(11):2208. doi: 10.3390/v13112208 (PMC8620829; doi:10.3390/v13112208)
Supplement: Supplementary file 1 [file viruses-13-02208-s001.zip › viruses-1418009-supplementary.pdf]

viruses

MDPI

Supplementary material

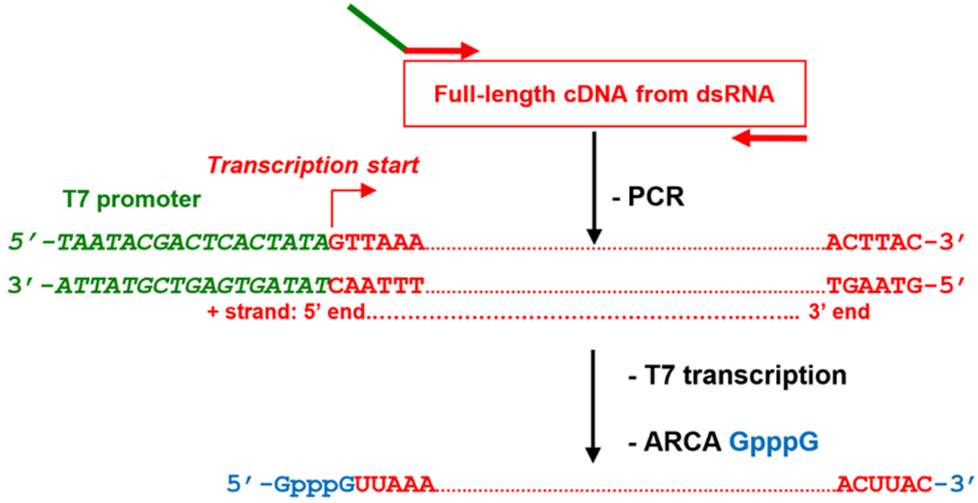

Figure S1. A schematic representation for generation of transcripts from linear PCR products representing the full-length cDNA copies of BTV genome segments (ARCA: anti-reverse cap analogue).

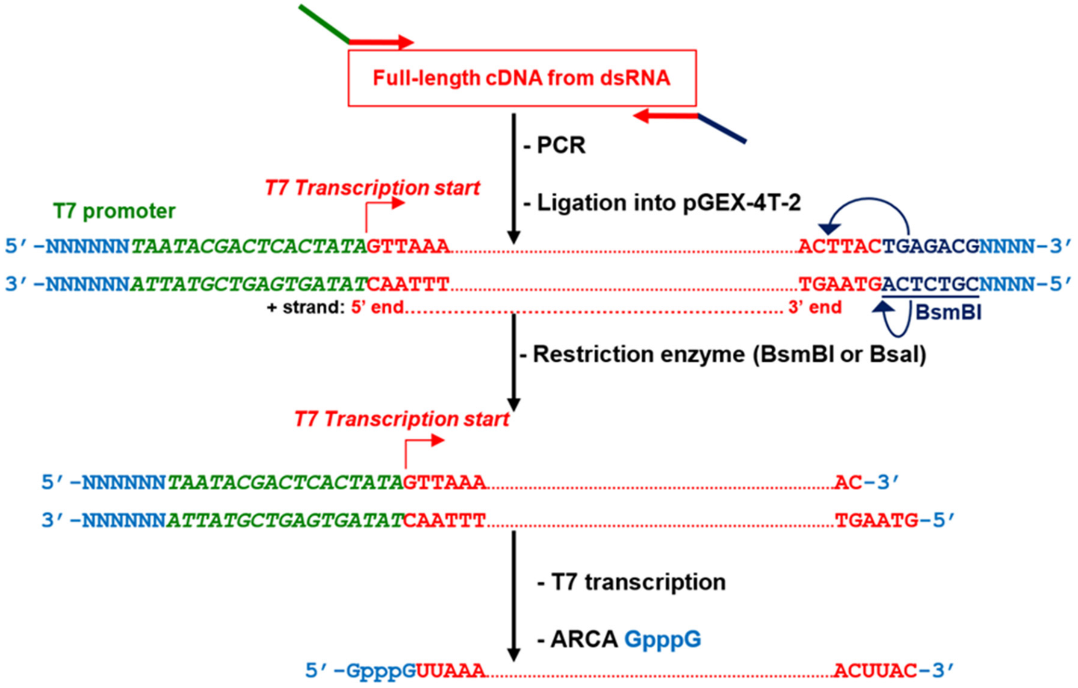

Figure S2. A schematic representation for generating transcripts from restriction enzyme linearised plasmids containing the full-length cDNA copies of BTV genome segments (ARCA: anti-reverse cap analogue).

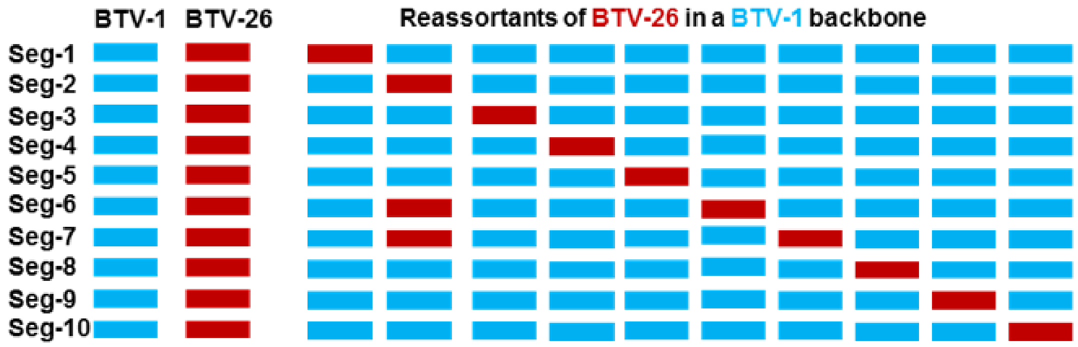

Figure S3. Schematic representation showing RNA combinations used to generate reassortants of BTV-26 in the BTV-1 backbone. The reassortants were designated RGBTV1:$X_{(26)}$ (e.g. RGBTV1:$1_{(26)}$, RGBTV1:$5_{(26)}$), where RGBTV1 refers to the genetic backbone of BTV1-RG_{C7}, and X refers to the specific genome segment of BTV-26 introduced into the reassortant.

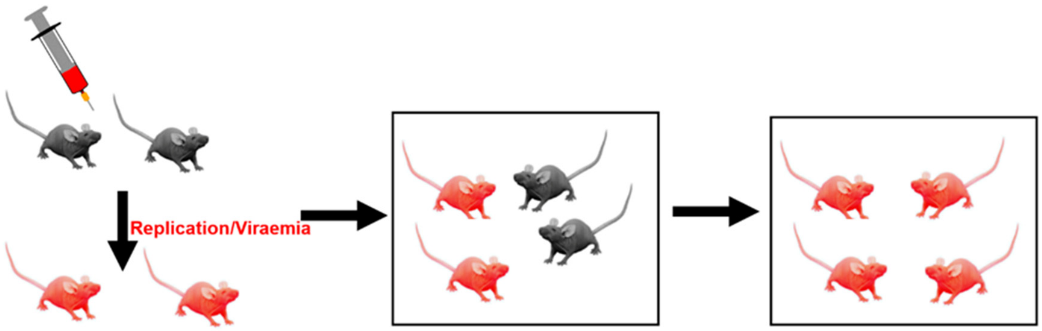

Figure S4. A schematic representation of the horizontal transmission experiment in $IFNAR^{(-/-)}$ mice. Red-coloured mice indicate where replication of a given BTV was detected by real-time RT-PCR.

Table S1. Primers designed for PCR amplification of full-length cDNAs of genome segments 1-10 of BTV-1. The minimal T7 promoter in the forward primer is shown in bold.

Virus/segment	Primer sequence 5′→3′
BTV1-S1T7FOR	**TAATACGACTCACTATA**GTTAAAATGCAATGGTCGCAATC
BTV1-S1REV	GTAAGTGTAATGCGGCGCGTGC
BTV1-S2T7FOR	**TAATACGACTCACTATA**GTTAAAATAGTAGCGCGATGGATG
BTV1-S2REV	GTAAGTCTAATAGTGCGCGGATC
BTV1-S3T7FOR	**TAATACGACTCACTATA**GTTAAATTTCCGTAGCCATGGCTG
BTV1-S3REV	GTAAGTGTGTTCCCGCTGCCGC
BTV1-S4T7FOR	**TAATACGACTCACTATA**GTTAAAACATGCCTGAGCCACACG
BTV1-S4REV	GTAAGTTGTACATGCCCCCCTC
BTV1-S5T7FOR	**TAATACGACTCACTATA**GTTAAAAAAGTTCTCTAGTTGGC
BTV1-S5REV	GTAAGTTGAAAAGTTCTAGTAGAGTG
BTV1-S6T7FOR	**TAATACGACTCACTATA**GTTAAAAAGTGCGCCCTTAGCGAA
BTV1-S6REV	GTAAGTGTAAGTGCTTCCCGTCGC
BTV1-S7T7FOR	**TAATACGACTCACTATA**GTTAAAAATCTATAGAGATGGACA
BTV1-S7REV	GTAAGTGTAATCTAAGAGACGTTTG
BTV1-S8T7FOR	**TAATACGACTCACTATA**GTTAAAAAATCCTTGAGTCATGGAG
BTV1-S8REV	GTAAGTGTAAAATCCCCCCCTAACC
BTV1-S9T7FOR	**TAATACGACTCACTATA**GTTAAAAAATCGCATATGTCAGCTG
BTV1-S9REV	GTAAGTGTAAAATCGCCCTACGTCA
BTV1-S10T7FOR	**TAATACGACTCACTATA**GTTAAAAAGTGTCGCTGCCATGCT
BTV1-S10REV	GTAAGTGTGTAGCGCCGCATACCCTC
